# Involvement of Anti-Inflammatory and Stress Oxidative Markers in the Antidepressant-like Activity of *Aloysia citriodora* and Verbascoside on Mice with Bacterial Lipopolysaccharide- (LPS-) Induced Depression

**DOI:** 10.1155/2022/1041656

**Published:** 2022-09-22

**Authors:** Denise B. Gomes, Patrícia Z. Serpa, Daniela Miorando, Maria Eduarda D. C. Zanatta, Camila S. Carteri, Lincon B. Somensi, Larissa Venzon, Ana C. Santos, Tauani C. S. França, Luísa M. Silva, Walter A. Roman Junior

**Affiliations:** ^1^Postgraduate Program in Health Sciences, Community University of Chapecó Region, Chapecó 89809-900, SC, Brazil; ^2^Pharmacognosy Laboratory, Community University of Chapecó Region, Chapecó 89809-900, SC, Brazil; ^3^Postgraduate Program in Development and Society, University of Alto Vale do Rio do Peixe, Caçador 89500-000, SC, Brazil; ^4^Program in Pharmaceutical Sciences, Chemical Pharmaceutical Research Nucleus (NIQFAR), University of Vale do Itajaí, Itajaí 89809-900, SC, Brazil

## Abstract

*Aloysia citriodora* Palau is popularly used to treat nervous disorders. Experimental evidence has indicated that verbascoside (VBS) isolated from *A. citriodora* has pharmacological potential. In this study, we evaluated the antidepressant-like effects of a hydroalcoholic extract of *A. citriodora* (HEAc) and VBS against lipopolysaccharide- (LPS-) induced depressive-like behavior in mice. In the pretreatment protocol (performed to evaluate the preventive potential), mice were pretreated with HEAc (3, 30, or 300 mg/kg) or VBS (30 mg/kg) before the administration of LPS. In the posttreatment protocol (performed to evaluate the therapeutic potential), mice were initially administered LPS and were subsequently given HEAc (3, 30, or 300 mg/kg) or VBS (30 mg/kg). In both treatments, the mice were submitted to an open-field test and tail suspension test (TST) at 6 and 24 h after LPS administration. The posttreatment evaluation revealed that HEAc (30 or 300 mg/kg) and VBS produced an antidepressant-like effect, as indicated by a reduction in the time spent with no movement in the TST. Moreover, HEAc (30 or 300 mg/kg) was found to reduce interleukin-6 (IL-6) levels and N-acetyl-glycosaminidase activity in the hippocampus, increase glutathione (GSH) levels in the hippocampus and cortex, and enhance IL-10 in the cortex and, at a dose of 300 mg/kg, reduced myeloperoxidase activity in the cortex. Contrastingly, no comparable effects were detected in mice subjected to the pretreatment protocol. Administration of VBS similarly reduced the levels of IL-6 in the hippocampus and increased GSH levels in the cortex. Our observations indicate that both HEAc and VBS show promising antidepressant-like potential, which could be attributed to their beneficial effects in reducing neuroinflammatory processes and antioxidant effects in the central nervous system.

## 1. Introduction

Despite clinical advances in the treatment of neuropsychiatric disorders, mood disorders such as major depressive disorder (MDD) are still considered an important public health problem. MDD is characterized by depressed mood and/or anhedonia, which may be related to other behavioral changes and vegetative symptoms that compromise physical and mental health [1]. Furthermore, depression is a leading cause of disability and affects approximately 300 million people worldwide [2].

Although considerable attention has been focused on the multifactorial and heterogeneous disorder of depression, its etiology remains poorly understood [3]. Furthermore, it is well established that depressive conditions often occur in the absence of a psychobiological triggering factor, and there is evidence to indicate the involvement of somatic, genetic, and environmental factors in its genesis, particularly the disturbance of hypothalamic functions and neurotransmission [4,5]. The monoaminergic hypothesis is currently the most widely accepted molecular mechanism proposed to explain the etiology of depression. According to this pathophysiological proposal, depression may arise because of depletion and disturbance of the activity of neurotransmitters, mainly serotonin, norepinephrine, or dopamine, in the central nervous system (CNS). Thus, several pharmacological agents used to treat MDD target this pathway, such as tricyclic antidepressants (TCAs), monoamine oxidase inhibitors (MAOIs), serotonin and noradrenaline reuptake inhibitors (SNRIs), and selective serotonin reuptake inhibitors (SSRIs) [4]. However, this therapy is not effective in all patients, which is in part attributable to the heterogeneity of the etiological factors of MDD which remain little explored. Moreover, these medications can have significant side effects, including nausea, drowsiness, vertigo, tremors, and fatigue [6–8]. In addition, it is estimated that 50% of patients are refractory to the initial antidepressant treatment and approximately 30% fail to reach remission after several pharmacological attempts [9].

In recent decades, advances have, nevertheless, been made regarding our understanding of the psychological bases underlying MDD, with observations of brain circuits revealing abnormal information processing as well as the characterization of the cellular and molecular alterations associated with this disorder. These advances have been made possible, at least in part, using rodent models, which have facilitated the investigation of molecular targets, including those stress axis abnormalities and elevated neuroinflammation [10].

Neuroinflammation is an important immune defense mechanism induced in response to infectious agents or damaged cells within the CNS. However, excessive neuroinflammatory responses may result in neuronal damage that is observed in several neurodegenerative diseases and disorders, including depression [11]. In this context, accumulating evidence indicates that inflammation may play a pivotal role in the pathophysiology of depression [12–14]. It has also been suggested that depression is an inflammatory disorder characterized by increased levels of proinflammatory cytokines such as tumor necrosis factor (TNF), interleukin-1*β* (IL-1*β*), and IL-6 in several brain regions [15], and similar findings have been reported in experimental studies [16]. Consistent with these observations, it has been demonstrated that the blockade of IL-1 or the inhibition of transcription factor nuclear factor kappa B (NF-*κ*B) shows promise in preventing depression-like behavior in rodents [17, 18].

The findings of other investigations have indicated that depressive patients generally show evidence of oxidative stress [19], which may arise from the continued disruption of the antioxidant defense system and an enhanced redox imbalance that culminates in an increase in the activity of reactive oxygen species (ROS) generation [20]. This perturbation of the balance between the system of oxidizing agents and antioxidants may lead to irreversible changes and promote tissue damage in different organs. Among the various organs, the brain is particularly susceptible to oxidative damage. This susceptibility can be attributed to the fact that the brain utilizes large amounts of oxygen and the fact that neural cells contain high levels of lipids, including nonsaturated fatty acids, with which free radicals readily react [21]. In addition, the oxidative stress induced by excess ROS can in turn exacerbate the expression of inflammatory cytokines and aggravate the pathogenesis of various mood disorders. Indeed, the findings of previous studies have indicated that exposure to persistent oxidative stress contributes to enhancing the susceptibility to depression and that these effects can be mitigated by treatment with antioxidants [22].

In animal models, lipopolysaccharide (LPS, a bacterial endotoxin) is used to create a neuroinflammation-related model of MDD that is characterized by behavioral changes, including a reduced sucrose preference and an increase in despair-like behavior. These symptoms are associated with the increased brain expression of proinflammatory cytokines, including IL-1*β*, IL-6, and TNF-*α* [23–26]. Notably, the levels of these inflammatory mediators can be reduced by the administration of certain natural compounds that exert modulatory effects in several brain areas [27].

In this latter context, human populations have for long selected and used plants according to their therapeutic and preventive significance; this has been documented in various ethnopharmacological records [28, 29]. These phytochemical compounds have been shown to exercise anti-neuroinflammatory effects against LPS-activated microglia via different mechanisms [27], and, in recent years, there has been a growth in the number of pharmacological studies that have examined the properties of plant-based extracts, substances isolated from plants, and synthetic derivatives based on promising natural products [30].

*Aloysia citriodora* Palau (Verbenaceae) is a medicinal plant native to South America, with global distribution, and is considered to have a high bioactive potential. This shrub, which can grow to heights of up to 3 m, has leaves with an aromatic lemon-like odor; hence it is commonly referred to as lemon verbena [31]. Depending upon the country in which it is found, the plant is also variously known as cidró, cidrão, erva-luisa, or dulce-lima. Reports describing the traditional use of this species by the Inca culture dating back to the 17th century exist, indicating its ethnopharmacological importance [32]. The plant is popularly used in South America, North Africa, and south of Europe to treat fever, insomnia, and anxiety [33], as well as digestive disorders, and as a sedative and antispasmodic agent [34]. Moreover, traditional Mexican medicine has used this plant for sleep and stomach disorders, depression, and anxiety [35]. Its biological effects can probably be ascribed to its essential oil, which is comprised primarily of monoterpenes (geranial, neral, and limonene) and sesquiterpenes, and flavonoids and phenylpropanoids are present in the plant in large amounts [36]. These compounds have also been demonstrated to have antioxidant, anxiolytic, anticancer, anesthetic, antimicrobial, and sedative effects, both *in vitro* and *in vivo* [37].

However, despite its widespread popular use, there have, to the best of our knowledge, been no studies that have examined the neuroprotective and antidepressant potential of the extracts and compounds isolated from *A. citriodora*. Given this deficiency, we sought to evaluate the antidepressant-like effects of the hydroalcoholic extract of the plant and its major constituent, verbascoside, using a mouse model of depression and investigated the underlying modes of action.

## 2. Methods

### 2.1. Plant Material

The plant material of *A. citriodora* was collected in Chapecó (SC) (27° 05′ 37″ S and 52° 39′ 58″ W) in October 2018. The botanical identification was performed by Professor Adriano Dias de Oliveira, curator of the Herbarium of the Community University of the Region of Chapecó (Unochapecó), where a voucher was deposited (#3777).

### 2.2. Extract Production of *A. citriodora*

The samples (leaves) from *A. citriodora* were dried at 25 ± 5°C and pounded in a knife mill (CiemLab®, CE430), passed through a sieve (32 Mesh/Tyler, 500 *μ*m), identified, and stored with protection from light. The hydroalcoholic extract (HEAc) was produced through extractive method of maceration (5 days) using dry-milled leaves of the plant (400 g) and ethanol 70% (4 L). After filtration through Büchner funnel, the HEAc was concentrated using evaporation under reduced pressure (40°C), lyophilized, weighed (149.48 g; 37.37% yielding), and stored at −20°C.

### 2.3. Chemical Analysis

#### 2.3.1. Estimation of Total Flavonoid Content

The total flavonoid content was analyzed according to Woisky and Salatino [38] with modifications. Briefly, 1 mL of the HEAc (1000 *μ*g/mL, in MeOH) was added to 1 mL of AlCl3 2% and after 60 min the spectrophotometer readings were performed at 425 nm. The calibration curve was produced using quercetin (in methanol) as standard (10, 15, 20, 25, and 30 *μ*g/mL), and the readings were performed in triplicate. The quantification of flavonoids was determined in milligrams per gram of extract.

#### 2.3.2. Obtainment of Verbascoside

A sample of HEAc (50 g) was diluted with water (500 mL) followed by mechanical agitation (20 min). Subsequently, the resulting solution was transferred to a separating funnel and submitted to liquid/liquid partition successively with hexane and EtOAc (ten times per solvent, 500 mL each). After solvent evaporation, an aliquot (2 g) of the EtOAc fraction was submitted to liquid column chromatography using silica gel (0.063–0.200 mm; Merck®, Darmstadt, Germany) as the stationary phase, as well as eluents hexane and EtOAc (10 : 100 v/v, respectively) in increasing polarity up to 100% EtOAc as mobile phase. The nine subfractions obtained were similarly pooled by using thin-layer chromatography (TLC) with EtOAc : MeOH : H_2_O (100 : 13.5 : 10 v/v) as the mobile phase and analyzed using a UV/Vis spectrometer at 366 nm and revealed with H_2_SO_4_ (10% in methanol) followed by heating at 110°C (10 min). Subfraction 8 (0.289 g) was observed as a spot by the TLC analysis, yielding verbascoside (VBS), which was identified by spectroscopic techniques (^1^H NMR, ^13^C NMR, and ESI-MS).

#### 2.3.3. Mass Spectrometry Analysis (ESI-IT-MSn)

The direct flow infusion of the samples was performed on the Thermo LTQ-XL apparatus (IT-MS) linear ion trap analyzer equipped with electrospray ionization (ESI) source in negative mode (Thermo, San Jose, CA, USA). Stainless steel capillary tube at 280°C, spray voltage of 5.00 kV, a capillary voltage of −35 V, tube lens of−100 V, and a 10 *μ*L/min flow were used. Multiple-stage fragmentations (ESI-MSn) were performed using the collision-induced decomposition (CID) method against Argon for ion activation. The first event was a full scan mass spectrum to acquire data on ions in the range of *m*/*z* 154–2000. The second scan event was an MS/MS experiment performed using a data-dependent scan on the [M-H]^–^ molecules from the compounds of interest at a collision gas flow rate of 30%.

### 2.4. Free Radical Scavenging *In Vitro* Activity

The free radical scavenging potential was evaluated by the decrease of 2,2-diphenyl-1-picrylhydrazyl (DPPH) absorbance, according to Xie and Schaich [39]. The aliquots of HEAc, VBS, ascorbic acid and gallic acids, and quercetin (15–250 *μ*g/mL), or distilled water (negative control group), were mixed with DPPH methanolic solution (400 *μ*g/mL). Subsequently, the samples were incubated (5 min at 25°C), and the absorbance was read at 517 nm in triplicate. The values were interpolated into a standard curve of DPPH (0–60 *μ*M) and expressed as IC_50_ (the concentration of the sample required to inhibit 50% of radical).

### 2.5. Animals

Adult female Swiss mice weighing 25–30 g (8 weeks) were provided by Central Animal House of Unochapecó and maintained in propylene cages under standard laboratory conditions (12 h light/dark cycle, the temperature of 22 ± 2°C), with free access to food and water. Food was withdrawn 12 h before the experiments and water was provided ad libitum. Several studies performed in female rodents at different stages of the estrous cycle have shown no interference in the results [40, 41]. The experiments were conducted following ARRIVE guidelines and the National Council for Animal Experimentation (CONCEA) after approval by the Institutional Animal Ethics Committee of Unochapecó (protocol number 014/18).

### 2.6. Dose-Response Study

According to popular usage, *A. citriodora* preparations are usually made with 15 g of plant material (approximately 2 g of dry leaves) and 150 mL of water, administered orally 2 to 3 times a day [31]. The extractive solution was dried (in an oven, 100°C), and the dry residue obtained was weighed and expressed as a percentage of yield about the dehydrated plant (0.49%). Thus, there is 2.238 mg of dry residue in 450 mL. For an 80 kg person, this administration represents an intake of approximately 27.9 mg/kg/day. Consequently, HEAc was tested at 3, 30, and 300 mg/kg, ensuring appropriate doses on a logarithmic scale to verify possible dose-response efficacy adequately and achieve a higher dose and a lower dose.

### 2.7. Experimental Design and Drug Treatment

The assays conducted in this study were performed following procedures described by Müller et al. [42], with minor modifications. Initially, the animals (Swiss mice) were randomly divided into two treatment groups. One group was subjected to a pretreatment, which was performed to evaluate the preventive potential of the *A. citriodora* extracts, and the other was subjected to a posttreatment, designed to evaluate the therapeutic potential of these extracts. The animals within these two groups were further subdivided to receive one of the following treatments (*n* = 8 per group, gavage). The naive group (*N*) was orally administered distilled water plus Tween 80 and not exposed to LPS, whereas the vehicle group (Veh) was treated orally with distilled water plus Tween 80 and exposed to LPS. Among the four treatment groups exposed to LPS, mice in three groups were administered HEAc (3, 30, or 300 mg/kg, p. o), whereas those in the fourth group were administered verbascoside (VBS, 30 mg/kg; p. o). As a positive control group, we treated mice with fluoxetine (Flu, 30 mg/kg). In the posttreatment protocol, we also assessed a group that received HEAc (30 mg/kg, p. o) and was not exposed to LPS, which was named per se.

**Figure 1:**
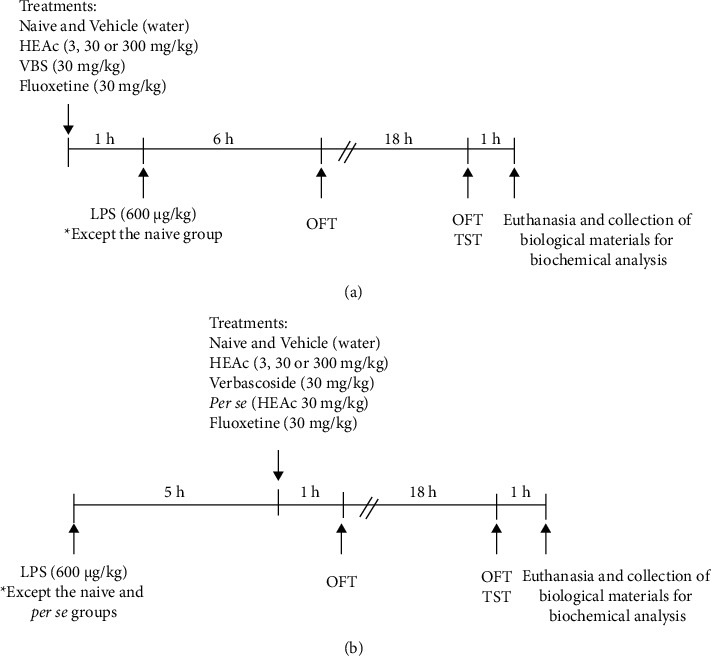
The timeline of the experimental design of the pretreatment (a) and posttreatment (b) stages in the evaluation of the antidepressant-like activity of the hydroalcoholic extract of *A citriodora* (HEAc) and verbascoside. Note: lipopolysaccharide (LPS; 600 *μ*g/kg, i.p). N (naive); Veh (vehicle, water); HEAc (*Aloysia citriodora* hydroalcoholic extract: 3, 30, or 300 mg/kg); VBS (verbascoside, 30 mg/kg); Flu (fluoxetine 30 mg/kg). OFT: open-field test; TST: tail suspension test.

In the preventive approach (pretreatment protocol, Figure 1(a)), which aimed to analyze the possible pretreatment effect of HEAc and VBS, the mice were acclimatized to the experimental room for 2 h, after which they were administered vehicle, HEAc, VBS, or fluoxetine (each (10 mL/kg) by oral gavage. After 1 h, all animals (except for those in the N and per se groups) received LPS (600 *μ*g/kg, i.p) [18], and, at 6 and 24 h after the administration of LPS, the mice were subjected to an open-field test (OFT) for 6 min. At 24 h after LPS administration, mice were subjected to a tail suspension test (TST) for 6 min.

In the posttreatment protocol (Figure 1(b)), except the N and per se groups, all groups received LPS (600 *μ*g/kg, i.p). At 5 h after the administration of LPS, the mice received the vehicle, HEAc, VBS, or fluoxetine by oral gavage, and, at 6 and 24 h after LPS administration, all animals were subjected to the OFT (6 min). At 24 h after LPS administration, the mice were evaluated in the TST (6 min).

At 1 h after the TST in both pre- and posttreatment protocols, all mice were anesthetized (thiopental sodium, 50 mg/kg; i.p) and subsequently euthanized by exsanguination. Thereafter the cortex and hippocampus were removed for subsequent analyses.

### 2.8. Behavioral Tests

#### 2.8.1. Evaluation of Sickness Behavior

Sickness behavior was characterized by the intensity of symptoms monitored at 15-minute intervals during the one hour before the OFT. Symptoms were assessed on a 4-point scale, as described by Gandhi et al. [43]. Mice were evaluated for lethargy (as indicated by reduced locomotion and a curled body posture), ptosis (drooping eyelids), and piloerection (ruffled, greasy fur), with each symptom being considered equivalent to 1 point, resulting in a scale ranging from 0 to 3, with zero being indicative of no symptoms and 3 indicating that all symptoms were present. Scoring was performed by three independent observers, and, after confirming the scores, the values were calculated as averages for subsequent statistical analysis.

#### 2.8.2. Open-Field Test (OFT)

The administration of LPS (600 *μ*g/kg, i.p) to animals is assumed to mimic the central and peripheral infectious processes in humans, resulting in adaptive responses. To determine the behavioral responses to the modulation promoted by the treatments, mice were evaluated for locomotor activity in the OFT at 6 and 24 h after LPS administration. The animals were placed individually in acrylic boxes (40 × 30 × 30 cm), the floor of which was divided into 24 equal squares. The crossing (number of squares crossed with the four paws), rearing (the number of times a mouse raised on its hind legs), grooming episodes (washing of the coat), and amount of fecal bolus were recorded in 6-min sessions.

#### 2.8.3. Tail Suspension Test (TST)

The activities of the HEAc, VBS, and fluoxetine on depressive-like behavior in mice before and after the LPS treatment were evaluated using the TST as previously described by Steru et al. [44], with minor modifications. Immediately after the OFT (24 h after LPS administration), the animals were suspended by the tail 70 cm above the floor using adhesive tape (1 cm from the tip of the end). During the final 4 min of each 6-min session, the time (in seconds) during which each mouse remained immobile was recorded.

### 2.9. Obtaining Homogenate of the Cortex and Hippocampus

This preparation was applied only for groups of animals that demonstrated an antidepressant-like effect on TSC in the pretreatment and/or posttreatment protocols. To analyze the markers in the total cortex and hippocampus, the tissues were homogenized in 200 mM phosphate buffer (pH 6.5). This homogenate was used to determine reduced glutathione (GSH), lipid hydroperoxides (LOOH), and N-acetyl-*β*-D-glucosaminidase (NAG). The supernatant was used to determine the activity of myeloperoxidase (MPO), superoxide dismutase (SOD), and catalase (CAT) enzymes, in addition to measuring the levels of cytokines (IL 6 and IL-10).

### 2.10. Evaluation of Inflammatory Parameters

#### 2.10.1. Determination of Myeloperoxidase (MPO) Activity

To quantify MPO activity, the pellets were resuspended in 80 mM potassium phosphate buffer (500 *μ*L, pH 5.4) containing 0.5% hexadecyltrimethylammonium bromide (HTAB). Subsequently, the solution was centrifuged at 11000 × *g* for 20 min at 4°C. The supernatant was analyzed at 620 nm in the presence of 7.5% H_2_O_2_ and 3,3′,5,5′-tetramethyl-benzidine (TMB, 18.4 nM) and expressed as millimeter optical density units (mOD)/mg of protein [45, 46].

#### 2.10.2. Quantification of Cytokine Levels

Cortex and hippocampal supernatants were used to estimate cytokine levels by enzyme-linked immunosorbent assay (ELISA). The number of aliquots was equalized by protein concentration. Aliquots (100 *μ*L at 0.20 mg/mL of protein) were used to measure interleukin-6 (IL-6) and IL-10 using R&D Systems® (Minneapolis, MN) mouse cytokine ELISA kits according to the manufacturer's instructions. Cytokine absorbance was measured by a microplate reader at 450 and 550 nm.

#### 2.10.3. Determination of N-Acetyl-*β*-D-Glucosaminidase (NAG) Activity

The activity of NAG is based on the hydrolysis of p-nitrophenyl-N-acetyl-*β*-D-glucosamine (substrate) by N-acetyl-*β*-D-glucosaminidase, releasing p-nitrophenol [47]. Samples containing 25 *μ*L of the supernatant or 25 *μ*L of distilled water (blank) were incubated with 100 *μ*L of citrate buffer (5 mM, pH 4.5) in the presence of the substrate (2.24 mM). The plates were incubated for 60 minutes at 37°C and the reaction was interrupted with glycine buffer (200 mM, pH 10.4) and measured in a spectrophotometer at 405 nm. Results were expressed as optical density unit (O.D.)/mg protein/hour.

### 2.11. Evaluation of Oxidative Stress Markers

#### 2.11.1. Determination of Free GSH Levels

Aliquots of homogenate of the cortex and hippocampus were mixed with trichloroacetic acid (12.5%) using vortex (10 min) and centrifuged (15 min at 4000 ×*g*) [48]. TRIS buffer (0.4 M, pH 8.9) and 5,5′-dithiobis (2-nitrobenzoic acid) (DTNB, 0.01 M) were added to the supernatant. Subsequently, the absorbance was measured at 415 nm in a microplate reader. Results were compared to a standard GSH curve and expressed in *μ*g/g tissue.

#### 2.11.2. Determination of Lipid Hydroperoxide (LOOH) Levels

Lipid hydroperoxides are highly reactive peroxidation products, thus being determined by Ferrous Oxidation-Xylenol Orange (FOX2) [49]. For these analyses, the cortex homogenate was mixed with 90% methanol by vertexing and centrifuged (20 min, 9000 ×g). The supernatant was added to the FOX2 reagent and incubated for 30 min at room temperature. Absorbance was evaluated at 560 nm and results were expressed in mmol/mg tissue.

#### 2.11.3. Determination of the SOD Activity

The analysis of SOD activity was performed by using a method to evaluate the ability of a solution to inhibit pyrogallol autooxidation [50]. For this, supernatant aliquot of 1 mM pyrogallol was added to 200 mM Tris HCl-EDTA (pH 8.5) and incubated for 20 min. After the reaction time, the absorbance was measured at 405 nm and the amount of SOD capable of inhibiting the autooxidation of pyrogallol by 50%, relative to the control, was defined as a unit (U) of SOD activity. The results were expressed as U/mg of protein.

#### 2.11.4. Determination of the CAT Activity

To measure CAT activity, the reactions were performed in the presence of cortex supernatant aliquots, 5 mM TRIS/EDTA buffer (pH 8.0), 30% H_2_O_2_, and ultrapure water. The decrease in absorbance was measured at 240 nm for 1 min. The results were expressed in *μ*mol of H_2_O_2_ consumed/min/mg of protein [51].

#### 2.11.5. Measurement of Protein Concentration

Protein concentration was measured in a spectrophotometer at 590 nm using the Bradford reagent (Amresco®), and bovine albumin (0.012–0.100 mg/mL) was used as a standard curve.

### 2.12. Statistical Analysis

Data were evaluated as normality by the Shapiro-Wilk test and Grubbs' test was used to detect outliers. Statistically significant differences between groups were calculated by the application of an analysis of variance (ANOVA) followed by the Tukey test using the software GraphPad version 8.00 for Windows (GraphPad Software, La Jolla, CA, USA). The values were represented by means ± standard error of the means (S.E.M) and *p* values less than 0.05 (*p* < 0.05) were used as the significance level.

## 3. Results

### 3.1. Phytochemical Analysis

#### 3.1.1. Quantification of Flavonoids

Analysis though of spectrometric UV/Vis using calibration curve the quercetin (10–30 *μ*g/mL; *y* = 0.0614*x* – 0.0384; *R*^2^ = 0.9973) showed high amount flavonoids in the hydroalcoholic extract of *A. citriodora* (11.6 mg/g; 1.2%).

#### 3.1.2. Isolation and Identification of Verbascoside

**Figure 2:**
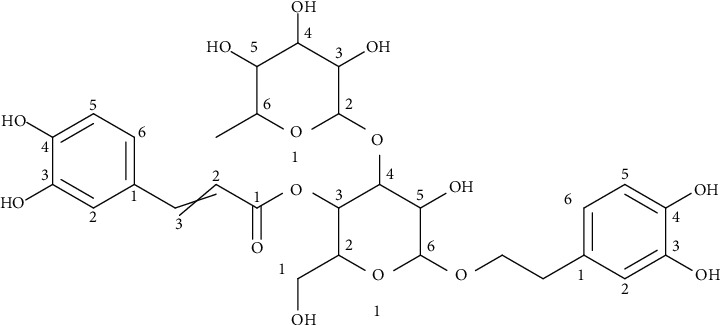
Chemical structure of verbascoside.

Verbascoside was isolated in subfraction 9 of fraction EtOAc of extract of *A. citriodora* (Figure 2). This compound was identified by comparison of their experimental spectra (^1^H NMR, ^13^C NMR, and ESI-MS) with those previously described [52–54]. Verbascoside or actoside: this compound was obtained as a crystalline powder, with a melting point of 142–145°C (water): (C_29_H_36_O_15_); ESI-MS: 623.08 [M+H]^−^; ^1^H NMR (400 MHz, CD_3_OD, *δ*, ppm): *δ* 1.09 (3H, *d*, *J* = 6.10 Hz, H-6″), *δ* 2.79 (2H, *m*, H-7), *δ* 3.28 (1H, *t*, *J* = 9.5 Hz, H-4″), *δ* 3.40 (1H, *t*, *J* = 9.0 Hz, H-2′), *δ* 3.55 (1H, *m*, H-5′, H-3″, H-5″), *δ* 3.52–3.72 (2H, *m*, H-6′), *δ* 3.73–4.05 (2H, *m*, H-8a, H-8b), *δ* 3.83 (1H, *t*, *J* = 9.0 Hz, H-3′), *δ* 3.93 (1H, dd, *J* = 3.3 Hz, *J* = 1.83 Hz, H-2″), *δ* 4.37 (1H, *d*, *J* = 7.89 Hz, H-1′), *δ* 4.93 (1H, *m*, H-4′), *δ* 5.19 (1H, *d*, *J* = 1.47 Hz, H-1″), *δ* 6.28 (1H, *d*, *J* = 15.97 Hz, H-8‴), *δ* 6.57 (1H, dd, *J* = 8.0 Hz, *J* = 2.00 Hz, H-6), *δ* 6.69 (1H, *d*, *J* = 8.00 Hz, H-5), *δ* 6.71 (1H, *d*, *J* = 2.02 Hz, H-2), *δ* 6.79 (1H, *d*, *J* = 8.00 Hz, H-5‴), *δ* 6.95 (1H, dd, *J* = 8.20 Hz, *J* = 2.0 Hz, H-6‴), *δ* 7.06 (1H, *d*, *J* = 2.02 Hz, H-2‴), *δ* 7.60 (1H, *d*, *J* = 15.90 Hz, H-7‴). 13C NMR (100 MHz, CD3OD, *δ*, ppm): *δ* 17,0 (C-6″), *δ* 36.5 (C-7), *δ* 62,4 (C-6′), *δ* 70.9 (C-4′), *δ* 71.0 (C-3″; C-5′; C-5″), *δ* 72 (C-2″), *δ* 72.4 (C-8), *δ* 73.9 (C-4″), *δ* 76.2 (C-2′), *δ* 81.7 (C-3′), *δ* 103.2 (C-1″), *δ* 104.4 (C-1′), *δ* 113.4 (C-8‴), *δ* 114.8 (C-2‴), *δ* 116.2 (C-5, C-5‴), *δ* 117.2 (C-2), *δ* 120.9 (C-6), *δ* 121 (C-6‴), *δ* 125.8 (C-1‴), *δ* 129.7 (C-1), *δ* 143.1 (C-4), *δ* 144.7 (C-3), *δ* 145.5 (C-3‴), *δ* 148.0 (C-7‴), *δ* 148.4 (C-4‴), *δ* 166.5 (C-9‴).

The negative mode mass spectrum (ESI-MS) of verbascoside (VBS) provided an *m*/*z* ratio of 623.08 revealing the deprotonated molecular ion [M–H]^−^, which confirmed the possible molecular mass as 624.51 compatible with the proposed molecule. Additional experiments on MS2 of the ion *m*/*z* 623 produced another ion from the main fragment at *m*/*z* 461. The ion at *m*/*z* 461 is considered to result from the loss of the caffeoyl moiety [M–H–162]^−^ of the parental ion *m*/*z* 623. The MS3 spectrum of the ion at *m*/*z* 461 yielded a very weak ion at *m*/*z* 315 upon losing a unit of rhamnose.

#### 3.1.3. *In Vitro* Assay of Free Radical Scavenging Activity

The antioxidant activities of HEAc, VBS (15–250 *μ*g/mL), and the positive controls (gallic acid, ascorbic acid, and quercetin) were determined based on the DPPH (2,2‐diphenyl‐1‐picryl‐hydrazyl‐hydrate) elimination assay. HEAc and VBS presented IC_50_ of 25.59 and 11.9 *μ*g/mL, respectively, confirming its strong antioxidant capacity *in vitro*. In line with expectations, gallic and ascorbic acids and quercetin also showed significant effects in reducing DPPH, with IC_50_ values of 3.27, 8.9, and 12.2 *μ*g/mL, respectively.

### 3.2. Behavioral Effects of the HEAc and VBS in the Pretreatment Experiment

**Figure 3:**
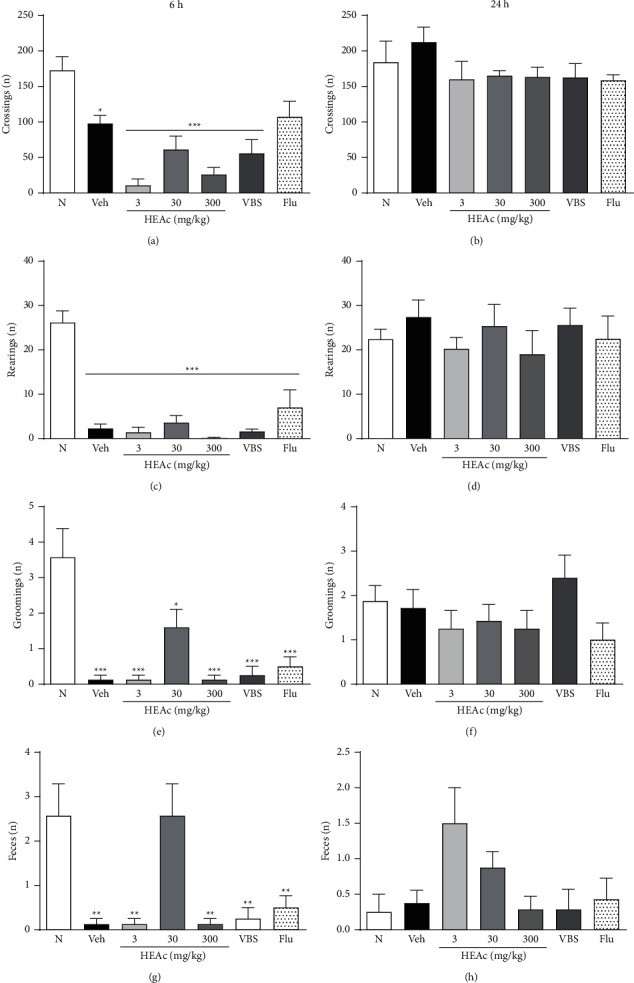
Behavioral effects of HEAc and verbascoside in mice in the pretreatment experiment. Note: pretreatment experiment: treatments administered (gavage) 1 h before exposure to bacterial lipopolysaccharide (LPS; 600 *μ*g/kg, i.p). N (naive); Veh (vehicle, water); HEAc (*Aloysia citriodora* hydroalcoholic extract 3, 30, or 300 mg/kg); VBS (verbascoside, 30 mg/kg); Flu (fluoxetine 30 mg/kg). A–H: values are expressed as mean ± SEM (*n* = 8). ANOVA (one way), post hoc Tukey. ^*∗*^*p* < 0.05, ^*∗∗*^*p* < 0.001, and ^*∗∗∗*^*p* < 0.001 compared to the naive group.

In the pretreatment experiment, we found that the administration of LPS 1 h after treatments was effective in promoting disordered behavior, mediated by inflammatory cytokines (sickness behavior). Accordingly, for the mice in all groups, we recorded maximum values of sickness behavior (4 points) characterized by lethargy (indicated by reduced locomotion and exploratory activity), hunched posture, ptosis, and piloerection. Subsequently, at 6 h after LPS exposure, mice in the vehicle group (exposed to LPS and treated with water, Veh) showed significantly reduced locomotor activity in the OFT compared with the mice in the N group (*p* < 0.05). Similarly, compared with the mice in the N group, we detected reduced locomotor activity in the mice administered HEAc (3, 30, or 300 mg/kg) or verbascoside (VBS; 30 mg/kg) when these were subjected to the OFT (*p* < 0.001). In contrast, mice treated with fluoxetine (Flu, 30 mg/kg) showed no significant difference compared with mice in both N and Veh groups (Figure 3(a)). Compared with the N group, we also observed a reduction in rearing and grooming among animals in the Veh, HEAc, VBS, and Flu groups which is probably due to the intensity of the sickness symptoms (*p* < 0.001) (Figures 3(c) and 3(e)). It is worth noting here that, at this stage of the experiment, except for the HEAc 30 mg/kg treatment group, all treatment groups showed a significant reduction in the number of feces compared with those produced by the N group mice (*p* < 0.01) (Figure 3(g)).

**Figure 4:**
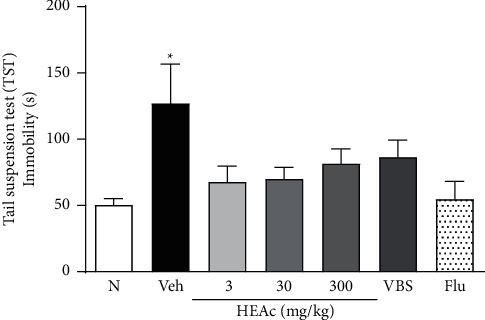
Antidepressant-like effects of HEAc and verbascoside (VBS) in the pretreatment experiment. Tail suspension test (TST) at 24 h after exposure to LPS. Note: pretreatment experiment: treatments administered (gavage) 1 h before exposure to bacterial lipopolysaccharide (LPS; 600 *μ*g/kg, i.p). N (naive); Veh (vehicle, water); HEAc (*Aloysia citriodora* hydroalcoholic extract 3, 30, or 300 mg/kg); VBS (verbascoside, 30 mg/kg); Flu (fluoxetine 30 mg/kg). Values are expressed as mean ± SEM (*n* = 8). ANOVA (one way), post hoc Tukey. ^*∗*^*p* < 0.05 compared to the naive group.

In line with expectations, we observed that, 24 h after exposure to LPS, all mice had recovered from the sickness behavior. Consequently, we observed no behavioral differences between mice in the different treatment groups and those in the N group (*p* > 0.05) concerning our evaluations of locomotor activity, rearing, grooming, and excretory behavior (Figures 3(b), 3(d), 3(f), and 3(h)). At this stage of the experiment, in the TST, the immobility times of the Veh group mice were longer than those of mice in the N group (*p* < 0.01), thereby confirming the depressive-like behavior in this neuroinflammation model. However, we detected no significant differences in this regard between mice in the treatment and Veh groups (Figure 4).

### 3.3. Behavioral Effects of the HEAc and VBS in the Posttreatment Experiment

**Figure 5:**
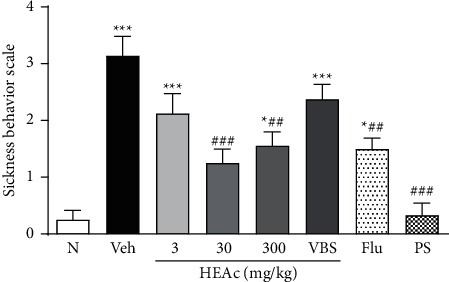
Intensity of sickness behavior of HEAc and verbascoside (VBS) in the therapeutic experiment. Note: N (naive); Veh (vehicle, water), hydroalcoholic extract of *Aloysia citriodora* (HEAc: 3, 30, or 300 mg/kg, po), VBS (verbascoside 30 mg/kg, po), Flu (fluoxetine 30 mg/kg), PS (Per se group, HEAc). Values are expressed as mean ± SEM (*n* = 8 mice/group). ANOVA (one way), post hoc Tukey. ^*∗*^*p* < 0.05 and ^*∗∗∗*^*p* < 0.001 compared to the naive group. ^##^*p* < 0.01 and ^###^*p* < 0.001 compared to the Veh group.

At 5 h after exposure to LPS in the posttreatment experiment, the mice in all groups were observed to show sickness behavior. Compared with the N group mice, those in the Veh group were found to be characterized by an increase in the intensity of symptoms (3.14 ± 0.34) (*p* < 0.001). However, compared with the Veh group mice, those in the HEAc (30 or 300 mg/kg) and Flu groups were observed to have lower intensities of sickness behavior (*p* < 0.001 and *p* < 0.01, respectively), thereby indicating a certain degree of protection against LPS (Figure 5).

**Figure 6:**
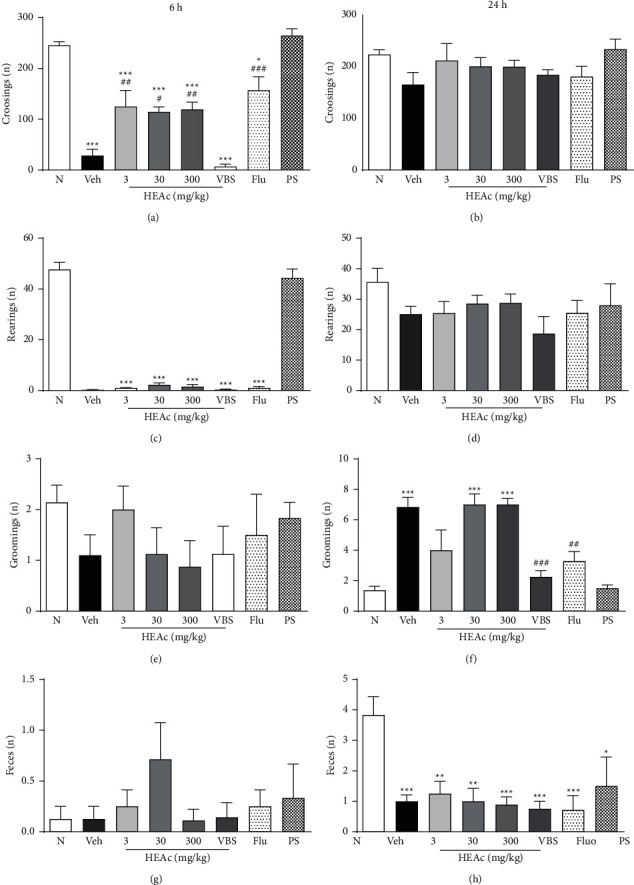
Behavioral effects of HEAc and verbascoside in mice in the posttreatment experiment. Note: posttreatment experiment: treatments administered (gavage) 5 h after exposure to bacterial lipopolysaccharide (LPS; 600 *μ*g/kg, i.p). N (naive); Veh (vehicle, water); HEAc (*Aloysia citriodora* hydroalcoholic extract 3, 30, or 300 mg/kg); VBS (verbascoside, 30 mg/kg); Flu (fluoxetine 30 mg/kg), PS (per se group, HEAc). A–H: values are expressed as mean ± SEM (*n* = 8). ANOVA (one way), post hoc Tukey. ^*∗*^*p* < 0.05, ^*∗∗*^*p* < 0.001, and ^*∗∗∗*^*p* < 0.001 compared to the naive group. ^#^*p* < 0.05, ^##^*p* < 0.001, and ^###^*p* < 0.001 compared to the Veh group.

After exposure to LPS for 6 h, we detected no significant differences between mice in the N and per se groups for the number of crossings in the OFT (245.30 ± 7.28 and 264.80 ± 13.61, respectively). However, compared with the N group mice, Veh group mice were characterized by a marked reduction in locomotor activity (*p* < 0.001), whereas mice receiving HEAc (3, 30, or 300 mg/kg) and Flu were observed to complete a significantly higher number of crossings compared with the Veh-treated mice (*p* < 0.01 and *p* < 0.001, respectively). Furthermore, mice administered VBS showed no significant difference in locomotor activity compared with the Veh group mice (Figure 6(a)). Concerning rearing, we observed that mice subjected to all treatments performed fewer rearing compared with the N group mice (*p* < 0.001), although we detected no significant difference between mice in the treatment and Veh groups. Moreover, with regard to the grooming and feces evaluations, we detected no significant difference among the N, Veh, and other treatment groups (Figures 6(c), 6(e), and 6(g)).

We observed that, 24 h after exposure to LPS, the mice in all experimental groups showed evidence of recovery from sickness behavior, as indicated by similar values in the numbers of crossings in the OFT. The treatment groups showed no significant difference from the N group in the amount of rearing (*p* > 0.05). However, compared with the Veh group, mice in the VBS and Flu groups showed less grooming behavior (*p* < 0.001 and *p* < 0.01, respectively), and the number of feces produced by mice in all treatments was lower than that produced by mice in the N group (*p* < 0.05) (Figures 6(b), 6(d), 6(f), and 6(h)).

**Figure 7:**
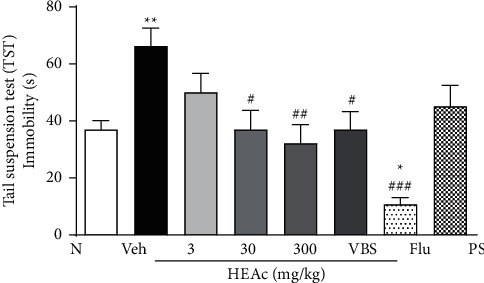
Antidepressant-like effects of HEAc and verbascoside (VBS) in the posttreatment experiment. Tail suspension test (TST) at 24 h after exposure to LPS. Note: posttreatment experiment: treatments administered (gavage) 5 h after exposure to bacterial lipopolysaccharide (LPS; 600 *μ*g/kg, i.p). N (naive); Veh (vehicle, water); HEAc (*Aloysia citriodora* hydroalcoholic extract 3, 30, or 300 mg/kg); VBS (verbascoside, 30 mg/kg); Flu (fluoxetine 30 mg/kg), PS (per se group, HEAc). Values are expressed as mean ± SEM (*n* = 8). ANOVA (one way), post hoc Tukey. ^*∗*^*p* < 0.05 and ^*∗∗*^*p* < 0.05 compared to the naive group. ^#^*p* < 0.05, ^##^*p* < 0.001, and ^###^*p* < 0.001 compared to the Veh group.

In the TST, the longest period of immobility (depressive-like effect) was recorded for mice in the Veh group (66.29 ± 6.31 s), which was significantly longer than that of the N group mice (*p* < 0.01) (37.01 ± 3.10 s). Results obtained for the per se group were found to be broadly comparable to those recorded for N group (45.17 ± 7.32 s), whereas, for mice administered HEAc at doses of 30 and 300 mg/kg and VBS, we detected significant reductions in the period of immobility compared with that of Veh group mice (*p* < 0.05, *p* < 0.01, and *p* < 0.05, respectively). As anticipated, mice receiving Flu showed the most pronounced treatment effect, with a marked reduction in the time spent with no movement compared with the Veh group mice (*p* < 0.001) (Figure 7).

### 3.4. Effect of HEAc and VBS on Inflammatory Markers in the Hippocampus and Cortex

**Figure 8:**
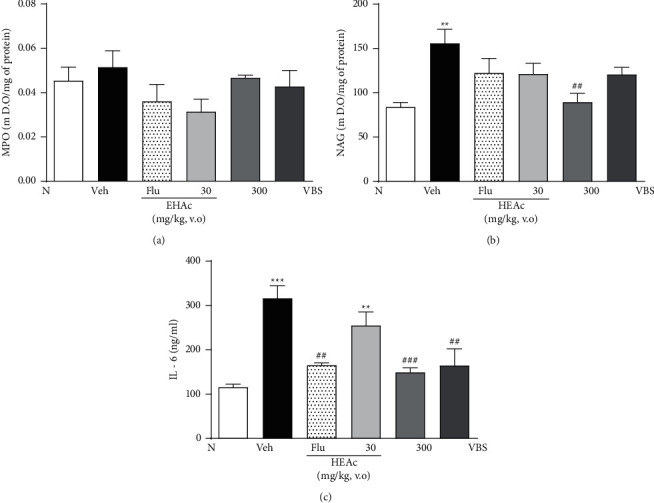
Effects of HEAc and VBS on the inflammatory markers MPO, NAG, and IL-6 in the hippocampus of mice evaluated in the posttreatment experiment of antidepressant-like activity. Note: N (naive); Veh (vehicle, water); Flu (fluoxetine 30 mg/kg); HEAc (*Aloysia citriodora* hydroalcoholic extract 30 or 300 mg/kg); VBS (verbascoside, 30 mg/kg). Values are expressed as mean ± SEM (*n* = 8). ANOVA (one way), post hoc Tukey. ^*∗∗*^*p* < 0.01 and ^*∗∗∗*^*p* < 0.001 compared to the naive group. ^##^*p* < 0.01 and ^###^*p* < 0.001 compared to the Veh group.

Due to the promising results obtained in the posttreatment experiment, the effects of HEAc (30 and 300 mg/kg) and VBS (30 mg/kg) (more effective doses in TST) on inflammatory markers in the hippocampus and cortex were investigated. In the hippocampus, treatments with HEAc (30 and 300 mg/kg), VBS, and Flu were not able to decrease MPO levels (Figure 8(a)). Elevated NAG levels in the Veh group (155.80 ± 15.75) compared (*p* < 0.05) to the N group (83.97 ± 5.09) were decreased by HEAc 300 mg/kg (42, 58%) compared to the Veh group (*p* < 0.01) (Figure 8(b)). The Veh group also increased the values of interleukin-6 (IL-6) (316.30 ± 28.16); however, HEAc (300 mg/kg) and VBS demonstrated a strong anti-inflammatory effect, decreasing IL-6 compared to the Veh group (47.13% and 47.94%; *p* < 0.001 and *p* < 0.01, respectively). Similar results to the fluoxetine (30 mg/kg) group were found (Figure 8(c)).

**Figure 9:**
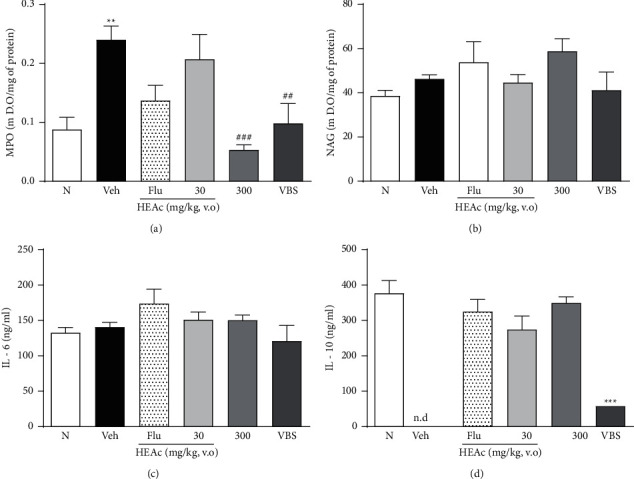
Effects of HEAc and VBS on the markers MPO, NAG, IL-6, and IL-10 in the cortex of mice evaluated in the posttreatment experiment of antidepressant-like activity.

In the cortex, the Veh group increased MPO levels (0.24 ± 0.02) in comparison (*p* < 0.01) with the N group (0.08 ± 0.02) and these levels were decreased by HEAc 300 mg/kg (77.91%; *p* < 0.001) and VBS (59.16%; *p* < 0.01), when compared to the Veh group (Figure 9(a)). No differences were observed between the groups tested in the levels of NAG and IL-6 (Figures 9(b) and 9(c)). In addition, the level of IL-10, an anti-inflammatory cytokine, was elevated in treatments with HEAc (30 and 300 mg/kg), with similar values in the Flu and N groups (Figure 9(d)).

### 3.5. Effect of HEAc and VBS on Markers of Oxidative Stress in the Hippocampus and Cortex of Mice Evaluated in the Posttreatment Experiment of Antidepressant-like Activity

LPS in the mouse hippocampus was able to decrease free GSH levels. In this evaluation, the N group had higher free GSH levels (24.18 ± 3.28) compared (*p* < 0.01) to the Veh group (11.52 ± 1.29). On the other hand, HEAc (30 and 300 mg/kg) prevented GSH depletion (85% and 213%, respectively) compared to the Veh group (*p* < 0.05 and *p* < 0.001). In the evaluations of SOD activity, no differences were observed between treatments and groups N and Veh (Table 1).

In the cortex, reduced levels of GSH (21.94 ± 0.84) were again observed in the Veh group, compared to the N group (36.97 ± 3.62) (*p* < 0.001), evidencing oxidative stress. However, HEAc at doses tested (30 and 300 mg/kg) as well as VBS restored GSH levels compared to group N. SOD and CAT activities as well as LPO levels were not significantly altered by HEAc and VBS, when compared with the N group (Table 2).

## 4. Discussion

Given that *Aloysia citriodora* Palau is widely used for its well-established culinary and ethnopharmacological properties [55], in this study, we sought to examine the purported antidepressant potential of this plant. Based on phytochemical analysis of the hydroalcoholic extract (HEAc) of the leaves of this plant, we were able to isolate the major constituent, verbascoside (VBS), and we subsequently evaluated the antidepressant-like activities of HEAc and VBS *in vivo*. Depressive behavior in mice was evaluated based on pre- and posttreatment administration of these agents in mice exposed to lipopolysaccharide (LPS), a bacterial endotoxin known to promote neuroinflammation. Our findings indicate that the pharmacological effects observed in mice administered HEAc (30 and 300 mg/kg) and VBS (30 mg/kg) may involve a reduction in inflammatory and oxidative parameters.

Our initial chemical analysis of HEAc revealed representative amounts of flavonoids consistent with the findings of Tammar et al. [56] who determined the presence of these compounds in the leaves of *A. citriodora* collected from different locations. Similarly, Skaltsa and Shammas [57] isolated several flavones from the plant extract, and, subsequently, Carnat et al. [58], Ragone et al. [59], and Quirantes-Piné et al. [52] detected the presence of flavone glucosides such as vitexin and isovitexin, in addition to apigenin-7-diglucuronide and chrysoeriol-7-diglucuronide. In more recent studies, further flavonoids, including jaceosidin, nepetin, and nepitrin, have been newly identified in this plant, all of which have a flavone structure [60]. Flavones are particularly active in the human body and can act in the CNS. More specifically, these compounds have been demonstrated to act on the benzodiazepine site in GABAA receptors [61, 62] and MAOA and MAOB enzymes [63], thereby providing clues as to their anxiolytic and anticonvulsant effects, as well as antidepressant activities [64]. In addition, HEAc fractionation used in this study provided isolation of VBS. Plants with known high concentrations of VBS have traditionally been used to treat inflammation and microbial infections [65]. These biological effects, along with the antioxidant, immunomodulatory, and neuroprotective activities of this compound, have previously been confirmed both in vivo and in vitro [66, 67]. This provides substantial evidence to indicate that the biological effects of *A. citriodora* can be attributed, at least in part, to the presence of flavonoids and VBS.

In recent years, numerous advances have been made in our efforts to gain a better understanding of the genetic, biochemical, and immunological changes involved in MDD. Recent investigations have, for example, demonstrated the interaction between proinflammatory cytokines and the hypothalamic-pituitary-adrenal (HPA) axis in patients with depression or experimental animals. Among the cytokines believed to be implicated in the association between stress and depression are IL-1*β* and IL-6, known to be potent activators of the HPA axis, which, consequently, can modulate noradrenergic and serotonergic mechanisms [68–70]. Furthermore, in animal models, cytokines have been shown to promote anhedonia, a key feature of depression [71]. They may also trigger disease-associated behavior (e.g., piloerection, ptosis, anorexia, and reduced social and exploratory behaviors) reflecting neurovegetative features of depression. Similar to cytokines, LPS is a potent activator of microglia and HPA, promoting neuroinflammation and depressive symptoms, respectively. In animal experiments, it has been established that the systemic administration of LPS promotes the activation of Toll-like-4 receptors (TLR4), thereby inducing the release of IL-1*β*, along with a cascade of other cytokines, including IL-6 and TNF. It is believed that these cytokines influence central neurons, resulting in certain behavioral changes [27, 72, 73].

In this study, we aimed to mimic the action of cytokines in neuroinflammation as well as the consequent behavioral responses. To induce a neuroinflammatory response, we administered LPS to mice according to two protocols that differed in terms of the temporal application of the treatments (either before or after treatment). In the pretreatment experiment, LPS administered to mice 1 h after treatments were observed to be highly effective in promoting sickness behavior. In line with expectations, 6 h after LPS exposure, all treatment groups showed a significant reduction in locomotor activity, as evaluated by OFT. We also observed reductions in rearing and grooming behavior and the production of feces, which we speculate could be attributable to an accentuation of the sickness behavior. However, 24 h after exposure to LPS, we found that all mice had recovered from the sickness behavior, and, in TST, only mice in the Veh group showed prolonged immobility, thereby corroborating the validity of this test. Based on these findings, we can thus conclude that, at the assessed doses, HEAc and VBS do not seem to have a protective effect against the adverse effects of LPS.

Although the findings of several studies have indicated the preventive effects of antidepressants or natural products on LPS-induced behavioral changes, similar therapeutic effects could not be demonstrated [74, 75]. In contrast, other authors have demonstrated that posttreatment administration of synthetic or natural compounds can mitigate LPS-induced depressive behavior in mice [18, 76, 77]. These seemingly contradictory observations serve to highlight the differences in the pharmacological effects between compounds as well as biological outcomes when using distinct experimental protocols [42].

In the posttreatment experiment, 5 h after exposure to LPS, the mice in all groups showed sickness behavior. However, we found that the intensity of depressive symptoms was lower in those mice administered HEAc (30 and 300 mg/kg) or fluoxetine (Flu), thereby providing evidence for a certain protective effect of these treatments against cell damage. At 6 h after LPS administration, mice in the Veh group showed a marked reduction in locomotor activity in the OFT. In contrast, mice receiving HEAc (3, 30, or 300 mg/kg) or Flu treatments showed a significant increase in the number of crossings compared with those made by Veh group mice. Thus, it appears probable that, apart from the lowest dose assessed, HEAc may interfere with the immune response of mice triggered by LPS. We suspect this could be attributable to the modulation of receptor activation pathways, such as TLR4, or the transcription factors of genes that are activated and regulated via NF-*κ*B, which regulates the genes associated with the responses of the innate and adaptive immune systems [78]. In addition, flavonoids and phenylpropanoids such as VBS are known to preferentially bind to benzodiazepine GABAA receptors and act as partial agonists promoting anxiolytic effects [79]. These observations may be consistent with the greater locomotion of mice treated with HEAc in the OFT, as mice in the per se group showed no significant differences from the N group mice, thus ruling out a stimulation effect.

A notable observation was that, at 24 h after exposure to LPS, the mice began to show evidence of recovery, as indicated by the similar values obtained in the OFT for the mice in different groups. Among the mice subjected to TST, we found that, compared with Veh group mice, those administered HEAc (30 and 300 mg/kg) or VBS were characterized by reductions in the period of immobility. In this test, the time an animal remains immobile is taken to be indicative of its state of despair and is assumed to reproduce a condition corresponding to human depression. Consequently, a reduction in the period of immobility is considered indicative of an antidepressant effect [80]. This finding is of particular interest, given the significant correlation between the efficacy of antidepressants and the data obtained using this biological model [81].

It is well established that the administration of LPS can promote the development of depressive-like behavior, and it has been proposed that this effect is associated with the induction of neuroinflammation in the cortex and hippocampus. Accordingly, preventing or ameliorating the inflammatory process could serve as an alternative approach to obtaining an antidepressant effect [27, 82–85].

Our findings provide evidence to indicate a certain effect of HEAc against neuroinflammation, including a reduction in N-acetyl-glucosaminidase activity in the hippocampus of mice administered the 300 mg/kg dose of this extract. This indicates that the treatment might protect cells against inflammation, as macrophages are also involved at the beginning of the inflammatory process [86]. Further, in the hippocampus, we observed that HEAc (300 mg/kg), VBS, and Flu showed an anti-inflammatory effect by decreasing IL-6 levels. IL-6 is among the main inflammation-associated cytokines and has also been shown to induce depressive-like symptoms in animal models of inflammation [87]. We detected a significant reduction in myeloperoxidase (MPO) activity in the cortex of mice treated with HEAc (300 mg/kg) or VBS. The activity of MPO is routinely monitored to evaluate the migration of neutrophils to the inflamed tissue, as a considerable amount of this enzyme is released into the extracellular medium following cellular activation [88]. In addition, we also detected elevated levels of the anti-inflammatory cytokine IL-10 in mice administered HEAc (30 and 300 mg/kg). Collectively, these observations provide persuasive evidence corroborating the anti-inflammatory effects of HEAc and its major constituent, VBS, in different regions of the brain. This is consistent with the proposition that a reduction in the inflammatory process is responsible, at least partially, for the observed antidepressant-like effects.

An additional mechanism that contributes to the anti-inflammatory activity of natural compounds is their ability to regulate the cellular redox state. This could be attributed to direct antioxidant action involving the capture of free radicals or by inducing the production of enzymes that comprise the endogenous antioxidant defense system (superoxide dismutase, catalase, and glutathione peroxidase) [89, 90]. In our oxidative stress evaluation tests carried out in the hippocampus, we established that administration of HEAc (30 and 300 mg/kg) prevented the depletion of glutathione (GSH), considered a first-line agent of antioxidant defense that plays a role in the capture of ROS [91]. This antioxidant effect of HEAc, mediated via the maintenance of basal levels of GSH, was similarly detected in the cortex of mice administered VBS. These observations are consistent with the intense antioxidant activities of HEAc and VBS observed in vitro, as indicated by the findings of DPPH radical scavenging assays.

## 5. Conclusion

In this study, we demonstrate that the hydroalcoholic extract of the leaves of *Aloysia citriodora*, along with its major constituent, VBS, has anti-inflammatory and antidepressant-like activities and propose that the pharmacological effects are mediated, at least in part, via a reduction in proinflammatory markers and oxidative stress. We accordingly believe that the findings of this study make an important contribution to the current understanding of the pharmacological mechanisms of this medicinal plant. In addition, our observations validate the widespread medicinal use of *A. citriodora* and offer the prospect of developing novel antidepressant agents.

## Figures and Tables

**Table 1:** Effect of HEAc and VBS on the levels of GSH and SOD activity in the hippocampus of mice evaluated in the posttreatment experiment of antidepressant-like activity.

Treatments	GSH	SOD
N	24.18 ± 3.28	22.24 ± 2.08
Veh	11.52 ± 1.29^*∗∗*^	18.10 ± 1.57
Flu	13.98 ± 0.64	25.30 ± 1.25
HEAc 30	21.34 ± 3.21^#^	20.09 ± 1.63
HEAc 300	36.06 ± 1.35^###^^*∗*^	19.32 ± 1.93
VBS	13.12 ± 1.00	20.24 ± 1.45

*Note.* N, naive; Veh, (vehicle, water); Flu, fluoxetine (30 mg/kg) hydroalcoholic extract of *Aloysia citriodora* (HEAc, 30 or 300 mg/kg); VBS, verbascoside (30 mg/kg). ANOVA (one way) (*n* = 8), post hoc Tuckey. ^*∗*^*p* < 0.05 and ^*∗∗*^*p* < 0.01 compared to the N group. ^#^*p* < 0.05 and ^###^*p* < 0.001 compared to the Veh group. Reduced glutathione (GSH; *μ*g/mg tissue); SOD (superoxide dismutase, U/mg protein).

**Table 2:** Effect of HEAc and VBS on oxidative parameters in the cortex of mice evaluated in the posttreatment experiment of antidepressant-like activity.

Treatments	GSH	LPO	SOD	CAT
N	36.97 ± 3.62	1.85 ± 0.03	43.23 ± 2.93	3.71 ± 2.69
Veh	21.94 ± 0.84^a^	1.85 ± 0.08	68.22 ± 2.18	6.89 ± 1.38
Flu	26.33 ± 2.84^a^	2.00 ± 0.06	41.75 ± 3.28^a^	3.91 ± 1.71
HEAc 30	36.39 ± 1.87^b^	2.02 ± 0.03	38.87 ± 3.05^a^	2.70 ± 1.02
HEAc 300	30.90 ± 1.24^b^	1.75 ± 0.08	64.40 ± 3.57	6.74 ± 0.29
VBS	30.81 ± 2.38^b^	1.94 ± 0.02	53.52 ± 2.43^a^	8.31 ± 1.58

*Note.* N, naive; Veh, (vehicle, water); Flu, fluoxetine (30 mg/kg) hydroalcoholic extract of *Aloysia citriodora* (HEAc, 30 or 300 mg/kg); VBS, verbascoside (30 mg/kg). ANOVA (one way) (*n* = 8), post hoc Tuckey. ^a^*p* < 0.05 compared to the N group (naive group). ^b^*p* < 0.05compared to the Veh group (vehicle group). Reduced glutathione (GSH; *μ*g/mg tissue); lipid hydroperoxide (LPO, *μ*m/MDA/g); SOD (superoxide dismutase, U/mg protein); CAT (catalase, *μ*mol H_2_O_2_/min).

## Data Availability

The articles, images, and analysis tables used to support the findings of this study are available from the corresponding author upon request.

## Abbreviations

MDD: Major depressive disorder
5-HT: Serotonin
NA: Norepinephrine
DA: Dopamine
ADT: Tricyclic antidepressants
MAOIs: Monoamine oxidase inhibitors
SSRIs: Selective serotonin reuptake inhibitors
LPS: Lipopolysaccharide from *Escherichia coli*
HEAc: Hydroalcoholic extract from *A. citriodora*
N: Naive
Veh: Vehicle
VBS: Verbascoside
PS: Per se
Flu: Fluoxetine
OFT: Open-field test
TST: Tail suspension test
GSH: Glutathione
LOOH: Lipid hydroperoxides
MPO: Myeloperoxidase
NAG: N-acetyl-*β*-D-glucosaminidase
SOD: Superoxide dismutase
GST: Glutathione transferase
CAT: Catalase
TNF-*α*: Tumor necrosis factor-alpha
IL-6: Interleukin-6
DTNB: 5,5′-Dithiobis (2-nitrobenzoic acid)
CDNB: 1-Chloro-2,4-dinitrobenzene
FOX2: Ferrous Oxidation-Xylenol Orange
CNS: Central nervous system
NF-ΚB: Nuclear factor Kappa *β* transcription
NOS-2: Nitric oxide synthase
COX-2: Cyclooxygenase-2
MAPK: Mitogen-activated protein
TLR: Toll-like receptors
STAT: Signal transducer and activator of transcription.
